# Cylindrospermopsin and Saxitoxin Synthetase Genes in *Cylindrospermopsis raciborskii* Strains from Brazilian Freshwater

**DOI:** 10.1371/journal.pone.0074238

**Published:** 2013-08-28

**Authors:** Caroline Hoff-Risseti, Felipe Augusto Dörr, Patricia Dayane Carvalho Schaker, Ernani Pinto, Vera Regina Werner, Marli Fatima Fiore

**Affiliations:** 1 Center for Nuclear Energy in Agriculture, University of São Paulo, Piracicaba, São Paulo, Brazil; 2 Faculty of Pharmaceutical Sciences, University of São Paulo, São Paulo, São Paulo, Brazil; 3 Natural Sciences Museum, Zoobotanical Foundation of Rio Grande do Sul, Porto Alegre, Rio Grande do Sul, Brazil; University of New South Wales, Australia

## Abstract

The *Cylindrospermopsis raciborskii* population from Brazilian freshwater is known to produce saxitoxin derivatives (STX), while cylindrospermopsin (CYN), which is commonly detected in isolates from Australia and Asia continents, has thus far not been detected in South American strains. However, during the investigation for the presence of *cyrA*, *cyrB*, *cyrC* and *cyrJ* CYN synthetase genes in the genomes of four laboratory-cultured *C. raciborskii* Brazilian strains, the almost complete *cyrA* gene sequences were obtained for all strains, while *cyrB* and *cyrC* gene fragments were observed in two strains. These nucleotide sequences were translated into amino acids, and the predicted protein functions and domains confirmed their identity as CYN synthetase genes. Attempts to PCR amplify *cyrJ* gene fragments from the four strains were unsuccessful. Phylogenetic analysis grouped the nucleotide sequences together with their homologues found in known CYN synthetase clusters of *C. raciborskii* strains with high bootstrap support. In addition, fragments of *sxtA*, *sxtB* and *sxtI* genes involved in STX production were also obtained. Extensive LC-MS analyses were unable to detect CYN in the cultured strains, whereas the production of STX and its analogues was confirmed in CENA302, CENA305 and T3. To our knowledge, this is the first study reporting the presence of *cyr* genes in South American strains of *C. raciborskii* and the presence of *sxt* and *cyr* genes in a single *C. raciborskii* strain. This discovery suggests a shift in the type of cyanotoxin production over time of South American strains of *C. raciborskii* and contributes to the reconstruction of the evolutionary history and diversification of cyanobacterial toxins.

## Introduction

The cyanobacterial genus *Cylindrospermopsis* (Woloszynska) Seenayya and Subba Raju [1] belongs to the order Nostocales, family Nostocaceae [2], [3]. To date, 10 species have been described, and all of them have been found in the phytoplankton community of freshwater environments [3]. *C. raciborskii* has been designated as the type species and the *C. raciborskii* strain AWT205, which was isolated from an ornamental lake (Oatley Pond) in Sydney, Australia [4], [5], as the recognized type strain. *C. raciborskii* has received attention in the last decade due to its frequent dominance in freshwater blooms and its ability to synthesize cyanotoxins. The production of cylindrospermopsin (CYN) and saxitoxin (STX) by this cyanobacterial species has been reported, but in distinct strains [6], [7]. Both toxins are synthesized on large modular non-ribosomal peptide synthetase (NRPS) and polyketide synthase (PKS) enzyme complexes [8]. CYN is a cyclic sulfated guanidine alkaloid that inhibits glutathione, cytochrome P450 and protein synthesis, causing injury and cell necrosis mainly in liver, kidneys, thymus and heart of vertebrates [9]–[11]. The gene cluster for the biosynthesis of cylindrospermopsin (*cyr*) in *C. raciborskii* strains spans 42-kb encoding 15 open-reading frames (*cyrA-O*) [12]. STX is also an alkaloid but acts as a neurotoxin that blocks neuronal sodium, potassium and calcium channels, affecting the propagation of nerve impulses resulting in neuromuscular paralysis [13], [14]. Thereby, these two cyanotoxins found in aquatic environments are a potential risk for human health.

Various strains of *C. raciborskii* are found colonizing eutrophic reservoirs in tropical, subtropical and temperate regions [15]–[19]. In Brazil, *C. raciborskii* is the main species found in freshwater bodies [7], [16], [20]–[31], although the occurrence of *C. phillipinensis*, *C. catemaco* and *C. acuminato-crispa* has also been reported [30], [32], [33]. The most studied Brazilian strain of *Cylindrospermopsis* is *C. raciborskii* T3, isolated in 1997 from the Taquacetuba branch of the Billings reservoir in São Paulo municipality [34]. Surprisingly, this strain was found to produce the neurotoxin saxitoxin and some of its derivatives [7]. Unlike the South American isolates, the *C. raciborskii* strains originated from Australia and Asia were found to synthesize the alkaloid cylindrospermopsin [6], [35]–[39]. The elucidation of the saxitoxin biosynthesis gene cluster (*sxt*) was based on the *C. raciborskii* T3 genomic DNA and comprises approximately 35-kb encoding for 31 open reading frames [40]. Chemical analyses conducted on other *C. raciborskii* Brazilian isolates also confirmed the production of saxitoxin and its analogues [7], [41], [42]. Although there are reports on the occurrence of CYN in environmental samples [43], [44], the production of this toxin by Brazilian *C. raciborskii* isolates has not been confirmed. In order to better understand this, four *C. raciborskii* strains were investigated for the presence of genes associated with the biosynthesis of CYN and SXT analogues. In this way, the *cyrA*, *cyrB*, *cyrC* and *cyrJ* genes, considered as being exclusives of CYN producers [12], [45], [46], were assessed, as well as *sxtA*, *sxtB* and *sxtC* genes. Both cyanotoxin groups were also evaluated by LC-MS analysis, and all strains were identified by both morphological and molecular analyses.

## Methods

### Cyanobacterial strains and morphological identification

The Brazilian cyanobacterial strains used in this study are shown in Table 1. The strains CENA302, CENA303 and CENA305 were isolated from water samples by transferring single *Cylindrospermopsis* trichomes using a sterilized Pasteur pipette to sterile test tubes containing 9 mL of ASM-1 [47] liquid medium. The trichomes were repeatedly transferred into new ASM-1 medium until a pure culture was established. Cycloheximide to a final concentration of 75 mg L^−1^ was added to inhibit eukaryotic cell growth. The cells were grown under a 14∶10 light-dark photoperiod with white fluorescent light (40±5 µmol photons m^−2^ s^−1^) at 25±1°C. Moreover, fluorescence microscopy studies (AxioSkop 2, Carl Zeiss, Jena, Germany, equipped with digital camera AxioCamMR3, AxioVision program, Rel. 4.6 software) of cyanobacterial cultures were also performed to confirm the absence of other cyanobacteria. Morphology was evaluated using reference literature [3], [48].

**Table 1:** Brazilian isolates of *Cylindrospermopsis raciborskii* used in this study.

Cyanobacterial strains	Origin	Date of sample collections
***C. raciborskii*** ** CENA302**	Riacho Grande branch, Billings reservoir, São Bernardo do Campo, SP23°46’33.9”S, 46°31’54.4”W	May 26, 2008
***C. raciborskii*** ** CENA303**	Theobaldo Dick lake, Lajeado, RS29°27'54.47"S, 51°58'15.51"W	July 3, 2009
***C. raciborskii*** ** CENA305**	Riacho Grande branch, Billings reservoir, São Bernardo do Campo SP23°46’33.9”S, 46°31’54.4”W	May 26, 2008
***C. raciborskii*** ** T3**	Taquacetuba branch, Billings reservoir, São Paulo, SP23°48’04.51”S, 46°37’35.41”W	May 7, 1997

The *C. raciborskii* strain T3 was isolated in 1997 by Dr. Pedro A. Zagatto (CETESB, São Paulo, Brazil) from Billings reservoir but in the Taquacetuba branch, São Paulo, São Paulo State, and was deposited in the culture collection of the Botanic Institute of São Paulo (CCIBt), Brazil. This strain was originally obtained from the CCIBt on June 4, 2003 and has since been maintained in culture in our lab. The Australian *C. raciborskii* strain CYP011K, originally isolated by Dr. Peter Baker from the Julius Lake, Mount Isa, Queensland, was obtained from the laboratory of Prof. Sandra M.F.O. Azevedo (Federal University of Rio de Janeiro, RJ, Brazil). This strain is known as a CYN producer and therefore was considered as a reference sample.

### DNA extraction, PCR amplification, sequencing and phylogeny

A total of 4.5 mL of cyanobacterial liquid culture was collected at the final exponential growth phase and concentrated by centrifugation (5 min at 13,000 × *g*). Total genomic DNA was extracted from the pellet using a modified CTAB (cetyl-trimethyl-ammonium bromide)-based extraction method adapted for cyanobacteria [49].

The presence of genes involved in the biosynthesis of cylindrospermopsin and saxitoxin were investigated using the specific primer sets (Table S1). All of the PCR reactions were performed in a 25 µL reaction volume containing 1X PCR buffer, 1.5 U Platinum® *Taq* DNA polymerase (Life Technologies, Carlsbad, CA, USA), 3.0 mM MgCl_2_, 200 µM dNTP, 0.2 µM of each primer and 10 ng of genomic DNA. Thermal cycling was performed in a Techne TC-412 Thermal Cycler (Bibby Scientific Limited, Stone, Staffordshire, England) using the designed primers (for *sxt* genes) under the following conditions: *sxtA4* (C-terminal domain), 94 °C for 5 min, followed by 35 cycles of 94 °C for 30 s, 61 °C for 30 s, 72 °C for 30 s, and final extension of 72 °C for 7 min; *sxtI*, 94°C for 5 min, followed by 35 cycles of 94°C for 30 s, 61 °C for 30 s, 72 °C for 90 s, and final extension of 72°C for 7 min; *sxtB*, 94 °C for 3 min, followed by 35 cycles of 94 °C for 30 s, 53 °C for 1 min, 72 °C for 1 min, and a final extension of 72 °C for 7 min. Thermal cycling conditions for c*yrJ* gene amplification were 94 °C for 3 min, followed by 30 cycles of 94 °C for 20 s, 53 °C for 1 min, 72 °C for 1 min, and a final extension of 72 °C for 7 min, adapted from Mazmouz *et al*., [50]. The *cyrA*, *cyrB* and *cyrC* fragments were amplified using the conditions previously described by the authors [51], with the exception of annealing temperature that was 57 °C.

The amplified gene fragments were cloned using pGEM®-T Easy Vector Systems (Promega, Madison, WI, USA). Competent *Escherichia coli* DH5α cells were transformed, and recombinant plasmids were purified from white colonies by the alkaline lysis method [52]. The cloned PCR products were sequenced using the DYEnamic ET Terminator Cycle Sequencing (GE Healthcare, Little Chalfont, Buckinghamshire, England) using the T7 and SP6 primer sites of the vector. The cycle sequencing reaction was performed with a Techne TC-412 (Bibby Scientific Limited) for 25 cycles of 95°C for 20 s, 52°C for 15 s, and 60°C for 1 min. After the completion of the reaction, a 75% isopropanol wash followed by a 70% ethanol wash was performed to remove residual dye terminators. The purified reaction mixtures were reconstituted in HiDi formamide (Applied Biosystems/Life Technologies, Foster City, CA, USA), and the samples were analyzed in an ABI PRISM 3100 Genetic Analyzer (Applied Biosystems/Life Technologies).

The 16S rRNA genes were PCR amplified using the specific primers listed in Table S1 and PCR amplification and sequencing were performed as described previously [53].

The partial nucleotide sequences of the *cyr* and *sxt* genes obtained in this study and reference sequences retrieved from GenBank were aligned, refined, and used to generate phylogenetic trees. Trees were reconstructed with maximum-likelihood (ML) and neighbor-joining (NJ) algorithms implemented by the MEGA version 5.0 program package [54] using the Tamura-Nei and ρ-distance models, respectively. The stability of the phylogenetic relationships was assessed by bootstrapping (1,000 replicates). Moreover, the gene sequences were analyzed by BLASTX (NCBI GenBank database) to compare each of the nucleotide sequence with its translated proteins in all possible reading frames. After identifying the correct reading frame, the nucleotide sequence was translated into protein using the translate tool from ExPASy Proteomic Server (Swiss Institute of Bioinformatics).

Nucleotide sequences generated in this study have been deposited in the NCBI GenBank database under the following accession numbers: JX175238 to JX175243 and KC894581 to KC894586 for the *cyr* genes; JX175232 to JX175237 and KC894587 to KC894589 for *sxt* genes; JQ707291 to JQ707296 for 16S rRNA genes.

### Chromatographic analyses

Culture samples of the *C. raciborskii* strains CENA302, CENA303, CENA305 T3 and CYP011K (20 days old) were filtered through Millipore glass fiber filters (Millipore, Milford, MA, USA) to harvest cells. Filters were frozen and later extracted with 0.05 M acetic acid by vortexing and cell disruption by sonication in a water bath for 15 min (2 cycles). After centrifugation (10,000 × *g* for 10 min), supernatants were recovered and filtered (0.45 µm, PVDF, Millipore) into appropriate vials. To assess the intra- and extracellular content of CYN and STX, 30 mL of homogeneous whole cultures were frozen and freeze-dried. After reconstitution and extraction in 1 mL 0.05 M acetic acid, samples were centrifuged (10,000 × *g* for 10 min) and filtered into appropriate vials.

Chromatographic analyses were performed with a Shimadzu Prominence (Kyoto, Japan) liquid chromatography system equipped with a diode array detector (SPD-M20A) and coupled to an ion trap mass spectrometer (Esquire HCT, Bruker Daltonics, Billerica, MA, USA). Separation was achieved at 0.2 mL min^−1^ in a Synergy Hydro column (150×2.0 mm, 4 µm; Phenomenex, CA, USA) under gradient elution of (A) water and (B) acetonitrile-water 90/10, both containing 5 mM ammonium formate and 0.01% formic acid. A linear gradient with a total run time of 35 min was used as follows: 1 to 30% solution B in 15 min, 30 to 90% B in 5 min, 90% B for 2 min, 90 to 1% B in 1 min and 1% B for 12 min. Absorption spectra were acquired in the 200-600 nm wavelength range. Detector effluent was split 1∶4 before entering the mass spectrometer through an electrospray source operated in the positive mode at 4 KV. Analyses were performed using nitrogen as nebulizing (35 psi) and drying gas (5 mL min^−1^, 300°C) and helium as buffer gas (4×10^−6^ mbar). Initial survey runs were taken by scanning the *m/z* range 50–800. In addition, multiple reaction monitoring (MRM) experiments using precursor ions at *m/z* 416 (CYN) and 400 (7-*deoxy*-CYN) were performed. For compound identification, product ion spectra were acquired and the fragmentation behavior analyzed according to Dörr *et al*., [55]. Because a standard solution of 7-*epi*-CYN was not available, efforts were not undertaken to separate this isomer, and therefore the single chromatographic band at 6.7 min for *m/z* 416 was assumed as CYN. A commercially available standard of CYN (Abraxis, Warminster, PA, USA) was also used for identity confirmation.

The saxitoxin analogues were investigated by three complementary analytical methods: two post-column oxidation methods with fluorescence detection (HPLC-FD), according to Diener *et al*., [56], [57], and a method based on hydrophilic interaction liquid chromatography coupled to mass spectrometry (HILIC-MS) following the recommendations of Soto-Liebe *et al*., [58]. Briefly, the compounds were separated on a Shimadzu Prominence (Kyoto, Japan) liquid chromatography system equipped with a post-column reaction oven and a fluorescence detector (RF-10AX). For HILIC-MS analyses, the chromatography system was coupled to an ion trap mass spectrometer (Esquire HCT, Bruker Daltonics, Billerica, MA, USA) through an electrospray ionization source. The identity of STX derivatives was confirmed by the fragmentation behavior of the [M+H]^+^ as well as the [M-H]^−^ ions [59]. Commercially available standards of STX derivatives (National Research Council/Institute for Marine Biosciences, Halifax, NS, Canada) were employed in HPLC-FD experiments for compound identification.

### Enzyme-linked immunosorbent assay (ELISA)

The presence of STX and CYN were tested with the saxitoxin and cylindrospermopsin ELISA kit (Abraxis LLC, PA, USA). All analyses were performed in accordance with the manufacturer’s instructions.

## Results

### Cylindrospermopsin (*cyr*) and saxitoxin (*sxt*) genes

**Figure 1:**
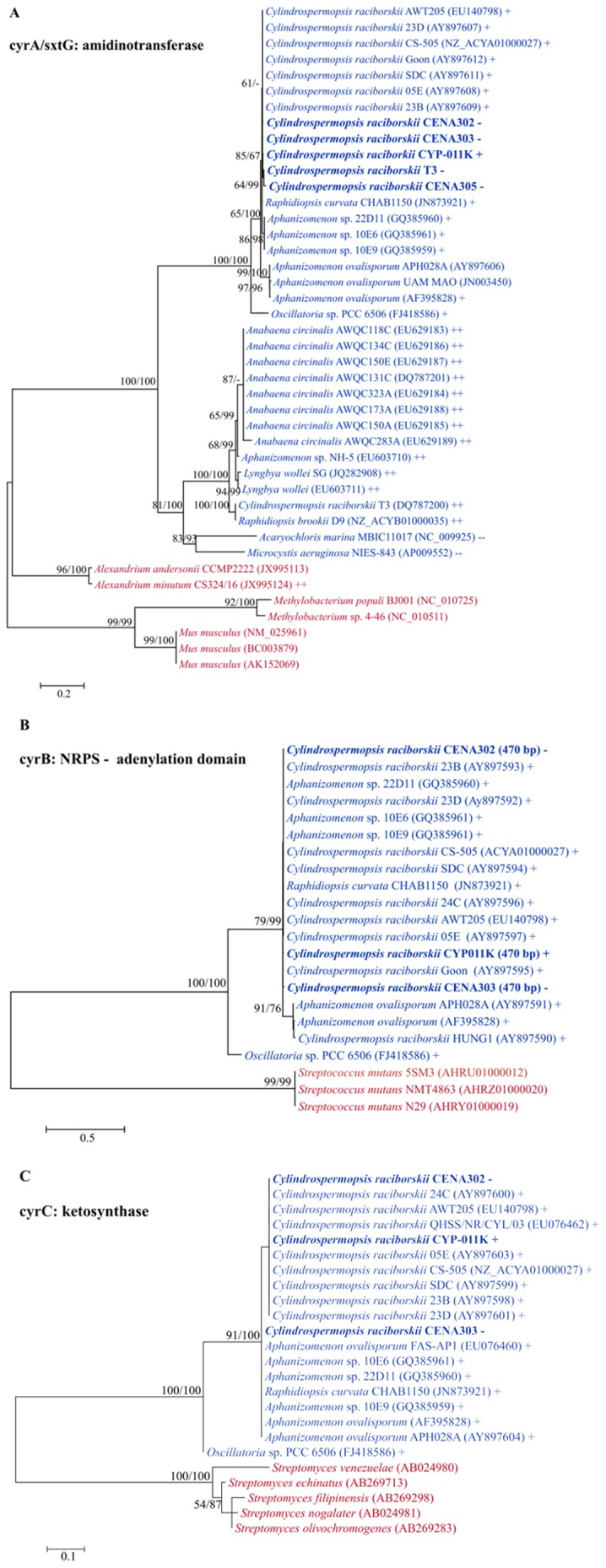
Maximum likelihood phylogenetic trees of *cyrA* (A), *cyrB* (B) and *cyrC* (C). The *C. raciborskii* Brazilian strains used in this study are shown in bold. Bootstrap test (1,000 resamplings) was performed and values >50% for ML and NJ analyses are shown over the nodes. Cyanobacterial taxa are shown in blue, other organisms in red. (+) CYN-producing and (–) CYN-non-producing strains; (++) STX-producing and (––) STX-non-producing strains.

The specific primer set (CYLAT-R/CYLAT-F), targeting the gene encoding the amidinotransferase enzyme involved in the biosynthesis of CYN, successfully amplified *cyrA* genes from the genomes of four Brazilian *C. raciborskii* strains (CENA302, CENA303, CENA305 and T3) and also from the Australian strain *C. raciborskii* CYP011K. These PCR products were sequenced and the almost entire *cyrA* gene sequences were obtained from the four Brazilian *C. raciborskii* strains (Table 2). However, the *cyrA* sequence of the T3 strain showed two nucleotide deletions in the position 525 and 1054 (Figure S1), causing a frameshift mutation. As a result, stop codons are replacing amino acid codons and hence the deduced protein sequence was truncated. The identities of the four *cyrA* sequences with homologous gene sequences from other CYN-producing closely related strains (*C. raciborskii* AWT205, *C. raciborskii* CS505, *C. raciborskii* CYP011K, *R. curvata* CHAB1150) were high and were larger than 98.8% (Table S2). However, lower identities were observed with *Aphanizomenon ovalisporum* (varying 95.5 – 95.8%) and *Oscillatoria* sp. strain PCC 6506 (varying 86.8 – 87.1%). In addition, the predicted protein functions and domains agreed with proteins from *C. raciborskii* AWT205 and CS505 (Figure S2), further supporting the identity of the sequences obtained as a CYN synthetase gene. The phylogenetic analysis grouped the *cyrA* sequences of *C. raciborskii* Brazilian strains together with other homologous nucleotide sequences from CYN-producing cyanobacteria with highly supported bootstrap value (100% ML and NJ) (Figure 1A). The amidinotransferase enzymes from other organisms grouped separately according to their biosynthetic pathway. Thus, sequences of the *sxtG* gene, that also encodes an amidinotransferase but is involved in the STX biosynthesis pathway, formed a fully supported clade distantly related to *cyrA* clade.

**Table 2:** Size of cylindrospermopsin and saxitoxin gene sequences obtained from the *Cylindrospermopsis raciborskii* strains.

	Cylindrospermopsin genes (bp)				Saxitoxin genes (bp)		
Cyanobacterial strains	*cyrA*	*cyrB*	*cyrC*	*cyrJ*	*sxtA*	*sxtB*	*sxtI*
*C. raciborskii* CENA302	1,106	470	515	–	201	336	904
*C. raciborskii* CENA303	1,106	470	515	–	200	270	589
*C. raciborskii* CENA305	1,106	–	413*	–	202	305	925
*C. raciborskii* T3	1,104	521*	413*	–	3,738**	957**	1,839**
*C. raciborskii* CYP011K	1,106	470	515	556	–	–	–

– no gene detected; * nonspecific sequences; ** complete sequences obtained from GenBank.

Three Brazilian strains (CENA302, CENA303 and T3) and also the Australian CYP011K strain showed positive results for the presence of a region of *cyrB* gene that encodes an adenylation domain of a NRPS. However, after sequencing the PCR products, the *cyrB* gene fragment obtained from the *C. raciborskii* T3 showed no identity with sequences deposited in the GenBank, an indicative of non-specific PCR amplification. The percentage of identity between partial *cyrB* adenylation domain of CENA302 and CENA303 was 99.4%, and ranged from 84.6 to 99.6% with other *cyrB* sequences retrieved from GenBank (Table S2). The phylogenetic analysis grouped the *cyrB* sequences of the two *C. raciborskii* Brazilian strains together with other homologous nucleotide sequences from CYN-producing cyanobacteria with highly supported bootstrap value (99% ML and NJ) (Figure 1B).

PCR products of a region of the *cyrC* gene that encodes a ketosynthase domain of a PKS were obtained from the genomes of the four *C. raciborskii* Brazilian strains as well as from the Australian CYP011K strain. However, after sequencing the amplicons, the sequences of the strains CENA305 and T3, with 99.0% identity between them, showed the highest identity (76.5% and 76.8%, respectively) with the phosphopantothenoylcysteine decarboxylase/phosphopantothenate-cysteine ligase of the *Anabaena* sp. 90. In the phylogenetic reconstruction of the partial *cyrC* sequences of the *C. raciborskii* Brazilian strains CENA302 and CENA303 a highly supported clade (bootstrap value of 99% ML and NJ) was formed with other homologous nucleotide sequences from CYN-producing cyanobacteria (Figure 1C).

Several attempts to amplify fragments of the *cyrJ* gene from all four Brazilian *C. raciborskii* strains were unsuccessful. Only in the Australian strain *C. raciborskii* CYP011K (CYN producer used as reference) partial *cyrJ* gene was successfully amplified.

**Figure 2:**
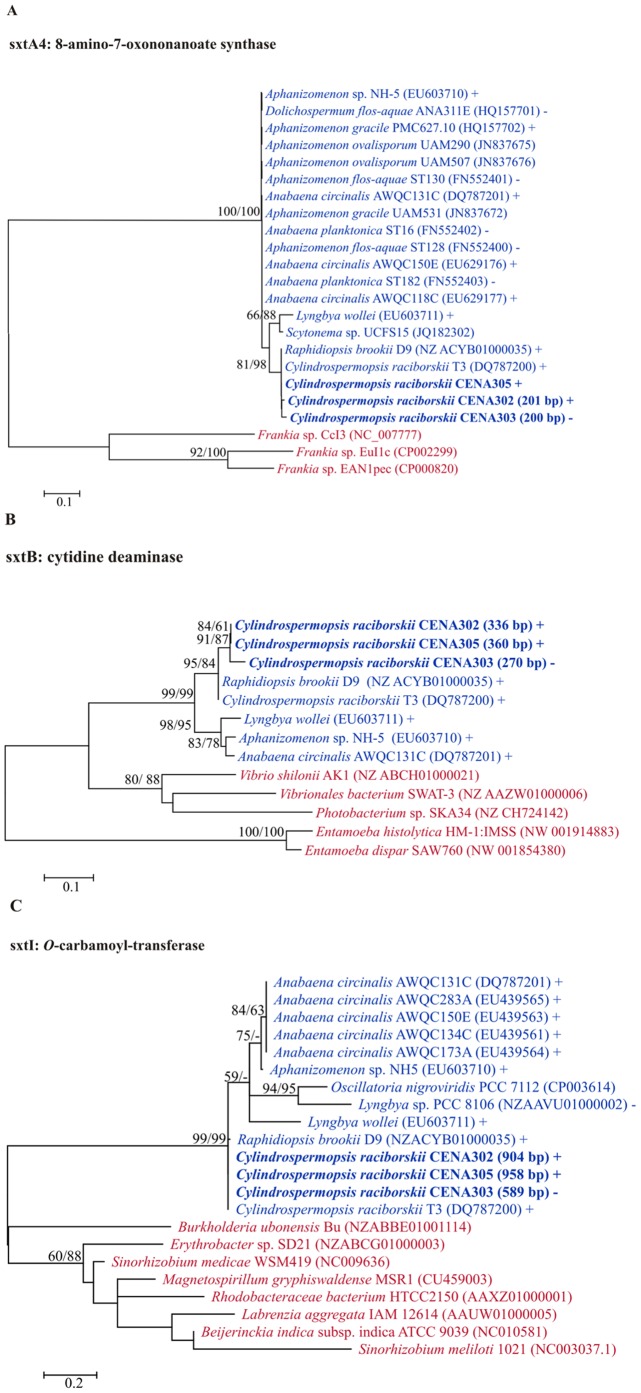
Maximum likelihood phylogenetic trees of *sxtA* (A), *sxtB* (B) and *sxtI* (C). The *C. raciborskii* Brazilian strains used in this study are shown in bold. Bootstrap test (1,000 resamplings) was performed and values >50% for ML and NJ analyses are shown over the nodes. Cyanobacterial taxa are shown in blue, other organisms in red. (+) STX-producing; (–) STX-non-producing strains.

The three *sxt* gene fragments (*sxtA4*, *sxtB* and *sxtI*) investigated were PCR amplified and sequenced from the genomes of almost all studied cyanobacteria, with the exception of the Australian CYN producer strain CYP011K, in which none of STX genes were detected (Table 2). BLAST analysis showed that the gene sequences of the *sxtA4* (8-amino-7-oxanonanoate synthase - AONS), *sxtB* (cytidine deaminase) and *sxtI* (*O*-carbamoyltransferase) from *C. raciborskii* CENA302, CENA303 and 305 strains had high similarity with the corresponding sequences from the STX-producing Brazilian strains *C. raciborskii* T3 and *Raphidiopsis brookii* D9 (Table S3). In the phylogenetic analysis, the *sxt* sequences clustered with other sequences of STX-producing cyanobacterial strains with supported bootstrap (100% ML and NJ) for *sxtA4*, (99% ML and NJ) for *sxtB* and *sxtI*, (Figure 2). The *sxt* gene sequences from T3 were identified previously [40]. The *sxt* genes were also compared with sequences that encode for homologous enzymes from other organisms and demonstrate phylogenetic differences according to its biosynthetic pathway. The predicted SxtA4, SxtB and SxtI protein functions and domains agreed with those homologous proteins from T3 and D9 (Table S3).

### Chemical and Enzyme Immunoassay analyses of CYN and STX

**Figure 3:**
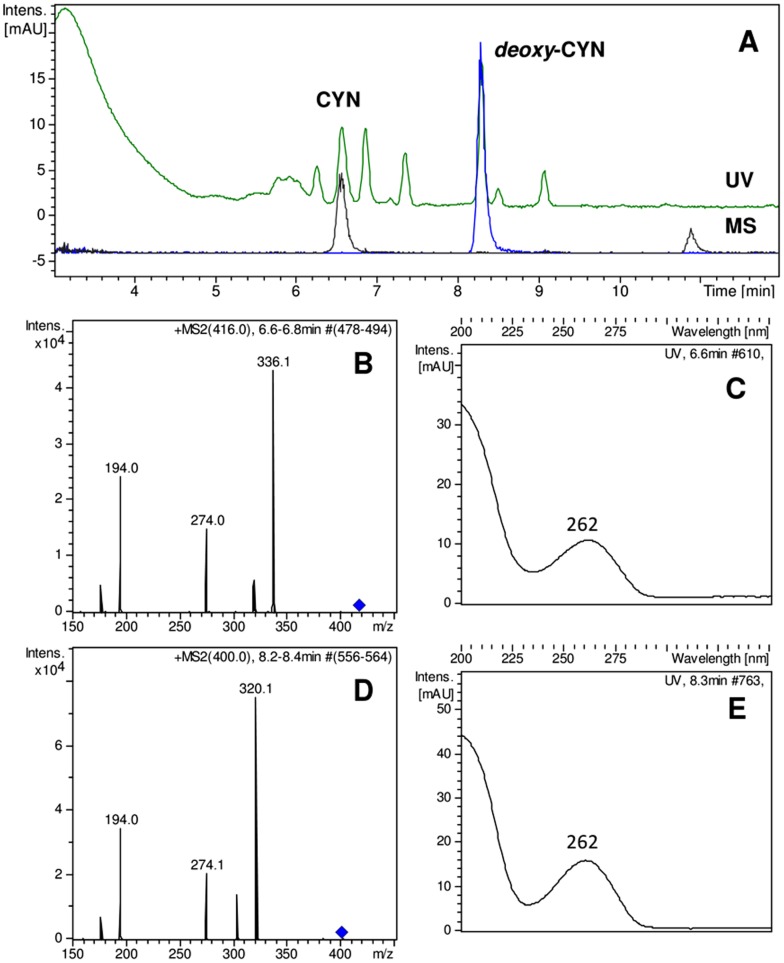
LC-UV-MS analysis of a freeze-dried culture sample of *C. raciborskii* CYP011K. (A) UV trace at 262 nm and extracted ion chromatograms for CYN (*m/z* 418) and 7-*deoxy*-CYN (*m/z* 400). Product ion spectra and UV absorption spectra are depicted for CYN (B and C) and 7-*deoxy*-CYN (D and E).

Despite several attempts to detect CYN and 7-*deoxy*-CYN derivatives in Brazilian strains, neither compound could be identified. As expected, both compounds were detected in the Australian CYP011K strain in the intra- and extracellular fractions (Figure 3). ELISA immunoassays were unable to detect CYN in the Brazilian strains of *C. raciborskii*, but showed positive results for Australian strain CYP011K. The lack of *cyrJ* amplification prompted the search for possible CYN derivatives missing the sulfate group at C-12, but all efforts failed to detect such a compound in the Brazilian isolates.

**Figure 4:**
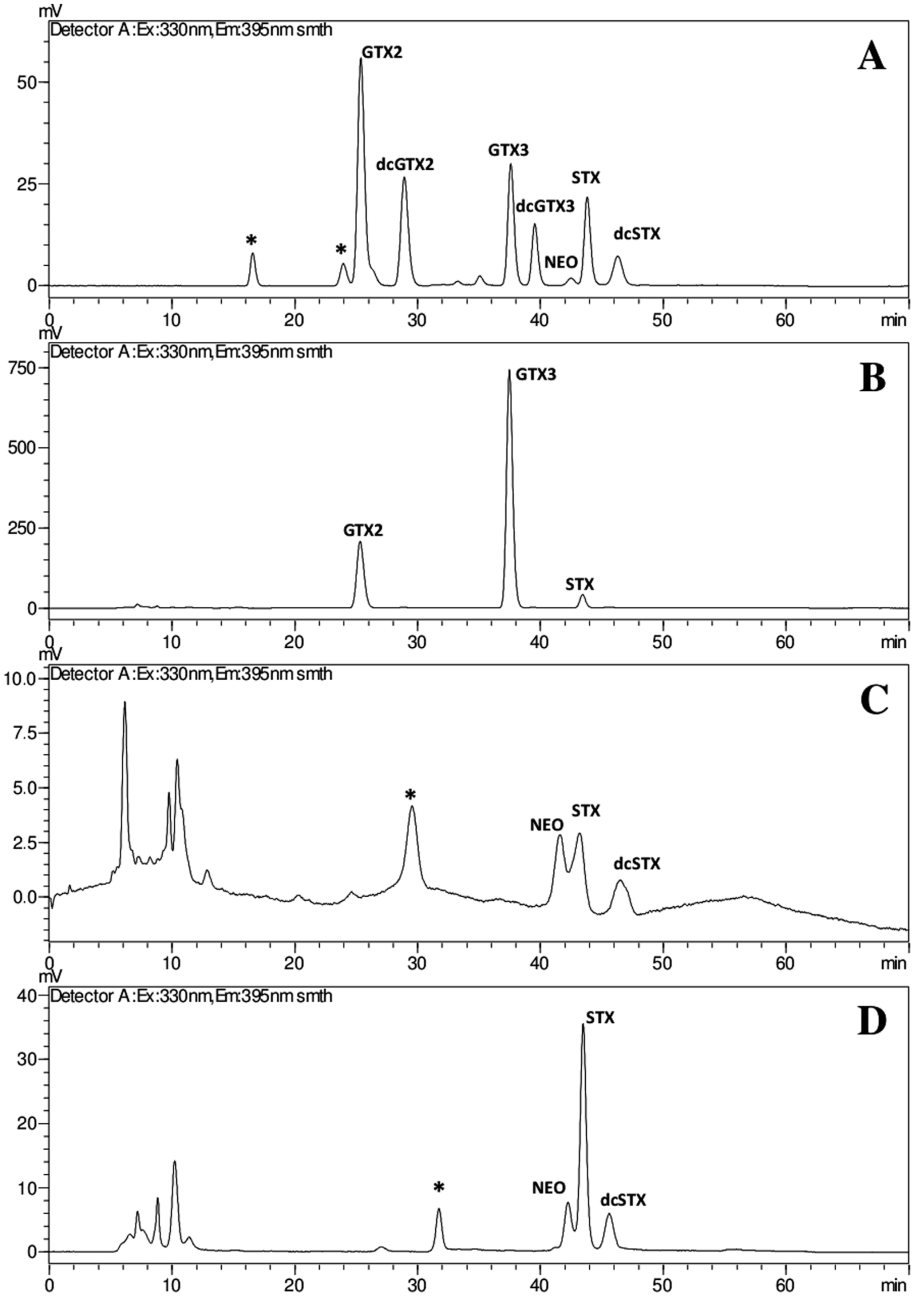
HILIC-FD chromatograms of STX variants in Brazilian *C. raciborskii* strains. (A) commercial standards; (B) *C. raciborskii* CENA302; (C) *C. raciborskii* CENA305; (D) *C. raciborskii* T3. Chromatograms acquired according to Diener et al. (2007). *denotes unspecific peaks.

Different chromatographic methods were employed to evaluate the production of saxitoxin analogues in the isolated strains. The post-column oxidation methods with fluorescence detection allowed the identification of the epimers GTX2/GTX3, STX and dcSTX in CENA302 (Figure 4A), STX, dcSTX, NEO and dcNEO in CENA305 (Figure 4B), as well as NEO, STX and dcSTX in strain T3 (Figure 4D). These results were further confirmed by HILIC-MS analyses. ELISA immunoassay provided positive results for STX in the strains CENA302, CENA305 and T3. None of the employed methods was able to detected STX analogues in the strains CENA303 and CYP011K.

### Morphological and 16S rRNA gene phylogenetic analyses

**Figure 5:**
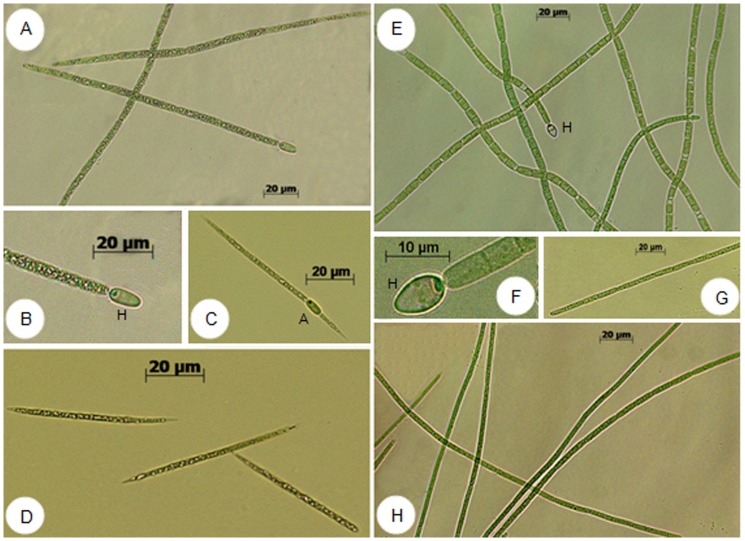
Brigthfield micrograph of *C. raciborskii* isolates showing details of trichome morphology, vegetative cells, heterocytous (H) and akinete (A). A-B) *C. raciborskii* CENA302; C-D) *C. raciborskii* CENA303; E-F) *C. raciborskii* CYP011K; G-H) *C. raciborskii* T3.

Morphological analysis of the isolated strains obtained in this study showed that they belong to the order Nostocales, genus *Cylindrospermopsis*. The filamentous strains presented typical characteristics of the *Cylindrospermopsis raciborskii* species (Figure 5) such as: subsymmetrical trichomes with cylindrical cells but attenuated and pointed at the ends of trichomes; pale blue-green or yellowish color; presence of aerotopes; conical terminal heterocytes developed after asymmetrical division of the end cells; and elongated oval akinetes situated adjacent to the heterocyte or the terminal vegetative cell [48], [3].

The nearly complete 16S rRNA gene sequences of *C. raciborskii* strains showed high similarity with sequences of *C. raciborskii* from GenBank (Table S4). The identities between the sequences of the four Brazilian *C. raciborskii* strains obtained in this study ranged from 99.1 to 100%. Moreover, these four 16S rRNA sequences displayed similarities varying between 99.2 to 99.6% with the sequence of the type strain *C. raciborskii* AWT205. The 16S rRNA sequences of the CENA302, CENA305 and T3 strains isolated from the Billings reservoir also showed high similarity (99.3%, 99.7% and 99.8%, respectively) with that of the *R. brookii* D9 found in the same reservoir. The 16 rRNA gene sequence of the *C. raciborskii* CYP011K showed 99.6 to 99.8% similarities with other sequences of *C. raciborskii* Australian strains (QHSS/NR/CYL/03, 05E and 23B).

**Figure 6:**
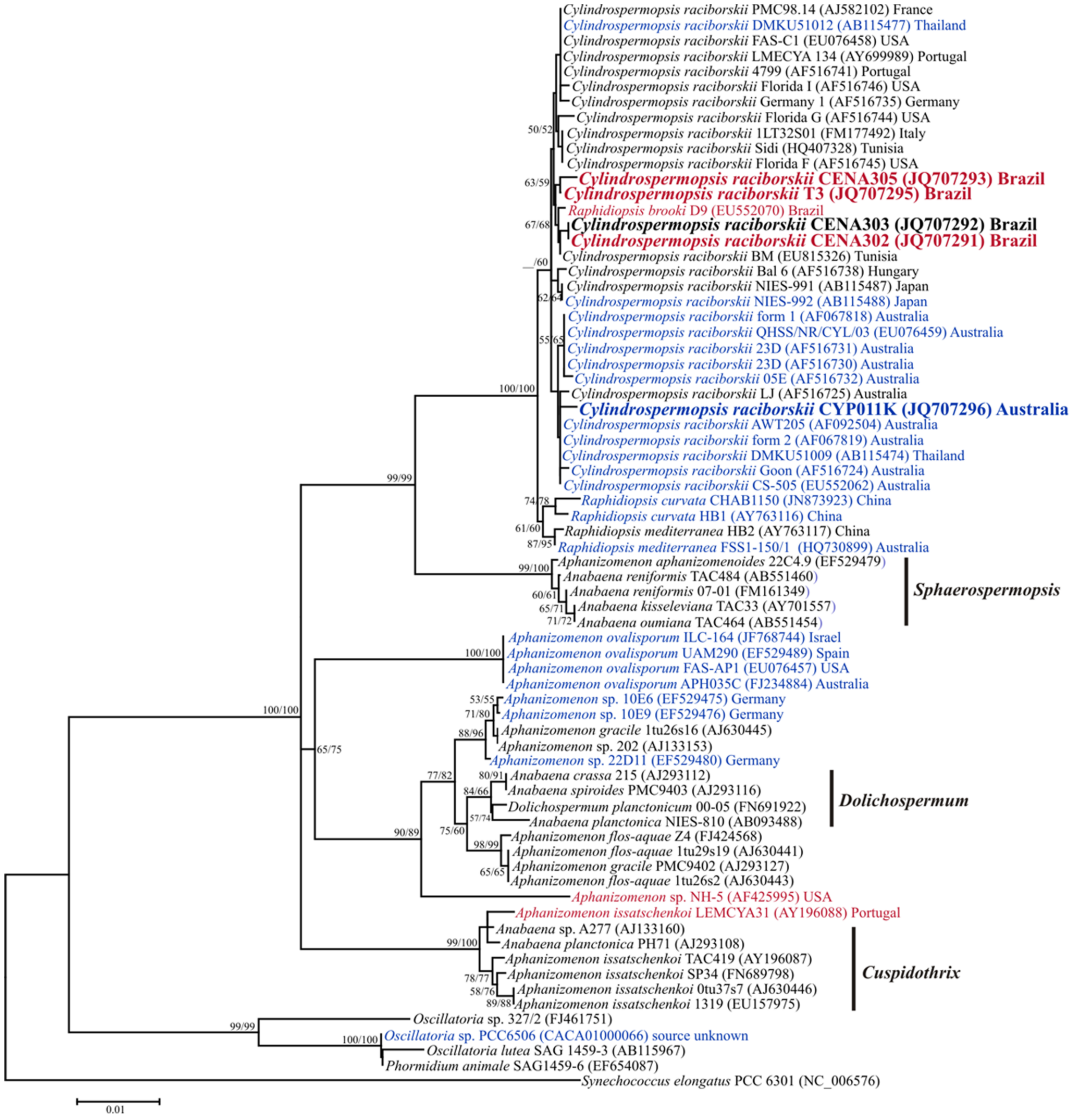
Maximum likelihood phylogenetic tree based on the 16S rRNA gene sequences showing the relationships of the studied cyanobacteria (in bold). Bootstrap test (1,000 resamplings) was performed and values >50% for ML and NJ analyses are shown over the nodes. Branch lengths are proportional to the number of substitutions per site (see scale bar). Taxon name in red or blue denotes STX or CYN producer strains, respectively.

In the phylogenetic tree, the 16S rRNA gene sequences of the *C. raciborskii* strains fall within a highly supported (bootstrap values of 99 and 100% for ML and NJ algorithms, respectively) major clade containing sequences of planktonic members of *C. raciborskii* isolated from several countries and an internal separated clade with members of *Raphidiopsis* genus (Figure 6). Within this major clade, the Brazilian and Australian strains formed distinct clades according to their origin but with low supported bootstrap. Strains of North American, African, European and Asian origin were mixed in other internal clades. The evolutionary relatedness of the four cyanobacterial strains (*C. raciborskii* AWT205, *C. raciborskii* CS-505, *Aphanizomenon* sp. 10E6 and *Oscillatoria* sp. PCC 6506) with the *cyr* clusters already described can be visualized in the phylogenetic tree. The phylogeny of the polyphyletic genus *Aphanizomenon* that possess several CYN and STX producer strains was also given in the phylogenetic tree. After revision of several genera of cyanobacteria, new designation for some of them was adopted and it is also shown in the phylogenetic tree.

## Discussion

In this study, cylindrospermopsin synthetase (*cyr*) genes were PCR amplified and sequenced for the first time from the genomes of four non-CYN-producing *C. raciborskii* strains isolated from Brazilian freshwater environments. Of the four *cyr* genes investigated and considered essential for the synthesis of CYN [12], [45], [46], three were amplified and sequenced from the genomes of two *C. raciborskii* Brazilian strains (CENA302 and CENA303). In other two Brazilian strains (CENA305 and T3) only the almost complete *cyrA* gene sequence was found. In addition, the four *cyr* genes were sequenced from the CYN-producing Australian strain *C. raciborskii* CYP011K. This Australian strain produces two CYN analogues, therefore must encode at least 11 genes (*cyrA, B, C, D, E, F, G, H, I, J* and *K*), that appear to be directly implicated in the synthesis of CYN according to the four *cyr* clusters already found in different cyanobacterial CYN-producing genera [60].

The almost complete *cyrA* sequences obtained from the four non-CYN-producing Brazilian *C. raciborskii* strains (CENA302, CENA303, CENA305 and T3) resemble those identified in the *C. raciborskii* strains AWT205 and CS-505 [12], [61]. The *cyrA* amplification product of T3 strain was achieved using more stringent annealing temperature than that recommended [51]. However, the truncated *cyrA* sequence of the T3 with the absence of two nucleotides indicated that this gene underwent reduction and was inactivated. The *cyrA* gene identified in the type strain *C. raciborskii* AWT205 is 1,176 bp long and encodes an L–arginine:glycine amidinotransferase enzyme responsible for the formation of guanidinoacetate and ornithine from L–arginine and glycine, the first step in the CYN biosynthesis [12], [46]. Amidinotransferases are a monophyletic group of enzymes distributed among vertebrates, plants and prokaryotes [46]. Furthermore, these enzymes are involved in biosynthesis of the neurotoxin saxitoxin in cyanobacteria (SxtG, L–arginine/L–lysine amidinotransferase) and dinoflagellates [40], [62]. Despite a high level of conservation with regard to residues involved in catalysis and substrate binding, these enzymes have different substrate specificities and kinetic mechanisms [63]. The CyrA amidinotransferase was shown to be unique to the metabolic pathway for biosynthesis of CYN [12], [46]. In this manner, the *cyrA* gene sequences obtained in this study formed a fully supported clade in phylogenetic analysis, together with other *cyrA* sequences of CYN-producing cyanobacterial strains retrieved from GenBank, but distantly related to the also fully supported clade containing *sxtG* sequences. This topology is in agreement with *cyrA* and *sxtG* phylogenetic reconstruction performed by Orr *et al*., [62], who defined the cluster containing the *cyrA* sequences as amidinotransferase 2 and that with *sxtG* sequences as amidinotransferase 1.

The CyrB enzyme (mixed NRPS-PKS) catalyzes the second reaction in the proposed biosynthetic pathway, incorporating an acetate unit into guanidinoacetate [12]. In the present study, the primer set used for detection of *cyrB* gene targeted the NRPS adenylation domain, which is responsible for amino acid recognition and activation. Therefore, this adenylation domain of CyrB uses the guanidinoacetate as a substrate for subsequent polyketide extensions. The two partial sequences of *cyrB* adenylation domain found in the genomes of two non-CYN-producing *C. raciborskii* Brazilian strains (CENA302 and CENA303) had high identities to other *C. raciborskii cyrB* adenylation domains and formed a fully supported clade in phylogenetic analysis. The partial *cyrB* amplification product of T3 strains was also achieved using more stringent annealing temperature than that recommended [51]. Nevertheless, the partial *cyrB* sequence obtained for T3 showed to be nonspecific amplification that led to a false-positive result. It is worth noting that a study applying neutrality test to the adenylation domain sequences of uncultured *A. ovalisporum*-like *cyrB* obtained from environmental samples indicated that it is under purifying selection. Purifying selection makes sure that deleterious mutations cannot take over a population and that any improved structures, once fixed in a population, are maintained as long as they are needed [64]. Furthermore, the *cyrB* gene of the CYN-producing *Oscillatoria* sp. PCC 6506 showed moderate identity (79%) to the one characterized in the toxic *A. ovalisporum* [50]. This *Oscillatoria* sp. PCC 6506 gene possesses a 150-bp-long GC rich fragment containing repeated sequences which encodes for a proline rich motif (PPLP) repetition that might function as a linker between the ketoreductase and methyltransferase domains of this NRPS-PKS hybrid enzyme. It is thus very likely that the *cyrB* from distinct cyanobacterial genera is evolutionarily related but that it substantially diverged from a common ancestor. The presence of *cyrB* sequence homologous to *cyrB* of the CYN producers was reported before for the non-toxic strain *C. raciborskii* Hung1 (Hungarian), however, *cyrA* and *cyrC* genes were not detected [51].

The CyrC, a polyketide synthase, is the subsequent enzyme proposed in the biosynthesis of CYN. It is responsible for the incorporation of a further acetate unit into guanidinoacetate, while a subsequent keto reduction provides the next intermediate [12]. The primer set used in this study targeted a region of the ketosynthase domain of the CyrC, which was highly similar to other *C. raciborskii cyrC* ketosynthase domain and formed a fully supported clade in phylogenetic analysis.

The *cyrJ* gene (780 bp long in the *C. raciborskii* AWT205) encodes a sulfotransferase, a tailoring enzyme responsible for the sulfation of the CYN [12]. The three structural variants of CYN described so far (CYN, 7-epi-CYN and 7-deoxy-CYN) are sulfated [65]-[67], therefore, the CYN-producing cyanobacteria must have the *cyrJ* gene. The lack of *cyrJ* gene amplification in all four Brazilian *C. raciborskii* strains indicates absence of this gene in the genomes of these cyanobacteria and supports the negative results of chemical analyses. These data also corroborated other studies indicating that *cyrJ* gene is only present in the genome of CYN-producing cyanobacteria [12], [50], [68]. It remains to be shown if the other nine genes (*cyrD, E, F, G, H, I, J* and *K*), that appear to be directly implicated in the synthesis of CYN, are present in these Brazilian *C. raciborskii* strains.

The importance of this finding is that, to date, only the saxitoxin genes have been reported in *C. raciborskii* strains isolated from Brazilian freshwater [40], [51] and this study demonstrates for the first time that Brazilian STX-producing *C. raciborskii* strains also carry fragments of *cyr* genes. These *cyr* genes could represent either a remnant or an otherwise ancestral intermediate of a functional CYN gene cluster. The acquisition/loss of cyanotoxin genes among cyanobacteria is not yet understood and vertical and horizontal gene transfer have been suggested for *cyr* and *sxt* gene clusters [50], [51], [60], [69]–[72]. These presumptions may be further elucidated with the increasing discovery of new cyanotoxin producers and their gene clusters.

The selective pressures in Brazilian environments that favored STX-producing *C. raciborskii* over CYN-producing strains are unknown. The physiological role of these secondary metabolites is still unclear, despite their effects on animal cells being relatively well understood. Recently, Soto-Liebe *et al*., [73] suggested STX analogues as protective compounds against elevated salt concentration in the environment. It remains to be demonstrated if STX variants produced by Brazilian *C. raciborskii* strains are replacing a possible cellular function of CYN.

Although the existence of CYN in Brazilian freshwater blooms has been known for several years, *C. raciborskii* strains that synthesize CYN have not been isolated so far. Thus, it is likely that other cyanobacterial genera are responsible for CYN production in Brazilian environments. In a similar way, no CYN-producing strains of *C. raciborskii* have been found in North America and Europe [74]–[76]. So far, in North America only *Aphanizomenon* strains were found to produce CYN [76], [77], while in Europe strains of *Aphanizomenon* [78], [79] and *Anabaena* [80] were identified as CYN-producers.

The presence of partial *sxt* gene sequences (*sxtA4*, *sxtB* and *sxtI*) in the CENA302, CENA303 and CENA305 strains with high similarities to those of other STX-producing strains already described, as well as the detection of some congeners by chromatographic analyses in the strains CENA302 and CENA305, corroborate previous findings that the Brazilian *C. raciborskii* strains produce these neurotoxins [7], [41], [42]. Moustafa and collaborators [70] showed that SxtA is comprised of two distinct regions and resulted from the fusion of two proteins acquired from different bacterial sources. The C-terminal region encodes an enzyme that presents significant identity to class I and II aminotransferases from actinobacteria. This region includes the SxtA4 catalytic domain and presents high similarity to AONS [40], [70]. Our phylogenetic analyses demonstrated that *sxtA4* gene sequences from cyanobacteria strains are highly conserved and different from the actinobacteria *Frankia* sp. suggesting that AONS from cyanobacteria is involved only in the STX biosynthesis.

The strain CENA302 was found to produce the epimers GTX2/GTX3, STX and dcSTX while CENA305 produces NEO, STX and dcSTX. Low levels of dcNEO (decarbamoyl neosaxitoxin) congener were detected only by HILIC-MS because co-elution hampered its identification in the HPLC-FD methods. A similar toxin profile was reported for *C. raciborskii* strains T2 and T3 [7]. Coincidentally, these four strains were isolated from the Billings reservoir in southeastern Brazil (subtropical climate) but at different locations. Our results of the STX toxin profile of *C. raciborskii* T3 are comparable to those obtained by Soto-Liebe *et al*., [58]. As noted by these authors, available literature on the toxin profile of T3 has been confusing given that several groups have identified different saxitoxin derivatives. In this study, the production of NEO, STX and dcSTX was confirmed by three complementary analytical techniques while dcNEO was detected only by HILIC-MS.

Previous phylogenetic analysis studies using nucleotide sequences of the fast-evolving 16S-23S internal transcribed spacer, *nifH* and *cpcBA*-IGS have shown a separation of *C. raciborskii* strains according to their geographic origin [81]–[83]. In our study, a phylogenetic tree based on the low-evolving sequences of 16S rRNA gene also showed a geographic separation of Brazilian and Australian strains according to their origin, but with low supported bootstrap. However, strains of North American, African, European and Asian origin were not geographically separated. If geographic isolation, rather than environmental selection, drives diversity, location-specific lineages would arise in different provinces regardless of microhabitat [84]. Nevertheless, studies on global biogeography of the cyanobacterial genera *Chroococcidiopsis* [84] and *Microcystis* [85] concluded that environmental selection drives diversification. Although some studies have shown geographic distribution of *C. raciborskii* strains, a larger number of nucleotide sequences from a variety of regions of different continents are needed to better understand the distribution and evolution of this cyanobacterial species.

## Conclusion

In this study, we identified and sequenced partial *cyr* and *sxt* synthetase genes of four Brazilian planktonic *C. raciborskii* strains, CENA302, CENA303, CENA305 and T3. Although the occurrence of both neurotoxins (anatoxin-a and homoanatoxin-a) and *cyr* genes has already been documented in a single *Oscillatoria* sp. strain [50], this is the first report to our knowledge of the presence of *sxt* and *cyr* genes in *C. raciborskii* strains. The results obtained here provide the first insight of the presence of CYN genes in *C. raciborskii* strains from an American country and contribute to the reconstruction of the evolutionary history and diversification of cyanobacterial toxins.

## Supporting Information

Figure S1 **Alignment of partial** ***cyrA*** **nucleotide sequences showing the two nucleotide deletions in the position 525 and 1054 (red marks) of the** ***Cylindrospermopsis raciborskii*** **T3.**
(TIF)

Figure S2 **Maximum likelihood phylogenetic trees of Cyr (A) and Sxt (B) amino acids sequences.** The *C. raciborskii* strains used in this study are shown in bold. Bootstrap test (1,000 resamplings) was performed and values >50% for ML and NJ analyses are shown over the nodes.
(TIF)

Table S1 **PCR primer sequences used in this study.**
(DOCX)

Table S2 **Percentage of identities of** ***cyr*** **nucleotide sequences of CYN-non-producing Brazilian strains of** ***C. raciborskii*** **with other sequences from CYN-producing cyanobacterial strains.**
(DOCX)

Table S3 **Percentage of identities of** ***sxt*** **nucleotide sequences of** ***C. raciborskii*** **Brazilian strains with other sequences from STX-producing cyanobacterial strains.**
(DOCX)

Table S4 **The 16S rRNA gene sequences identities among the Brazilian** ***C. raciborskii*** **strains and other sequences of related cyanobacterial strains.**
(DOCX)
