# Supplementary figures and images for: Cylindrospermopsin and Saxitoxin Synthetase Genes in Cylindrospermopsis raciborskii Strains from Brazilian Freshwater
Source: PLoS One. 2013 Aug 28;8(8):e74238. doi: 10.1371/journal.pone.0074238 (PMC3756036; doi:10.1371/journal.pone.0074238)

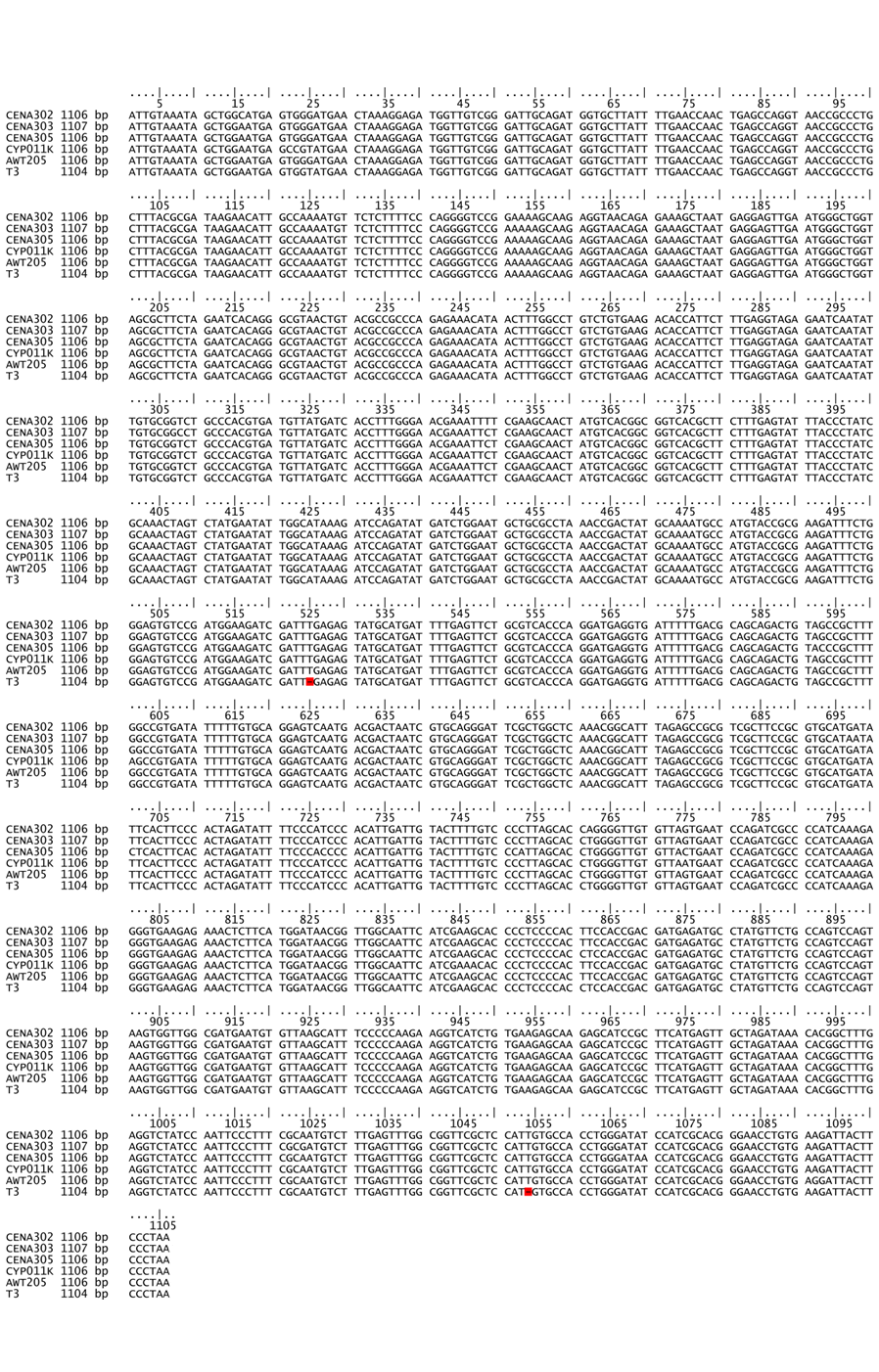

Supplement: Figure S1 — Alignment of partial cyrA nucleotide sequences showing the two nucleotide deletions in the position 525 and 1054 (red marks) of the Cylindrospermopsis raciborskii T3. (TIF) [file pone.0074238.s001.tif]

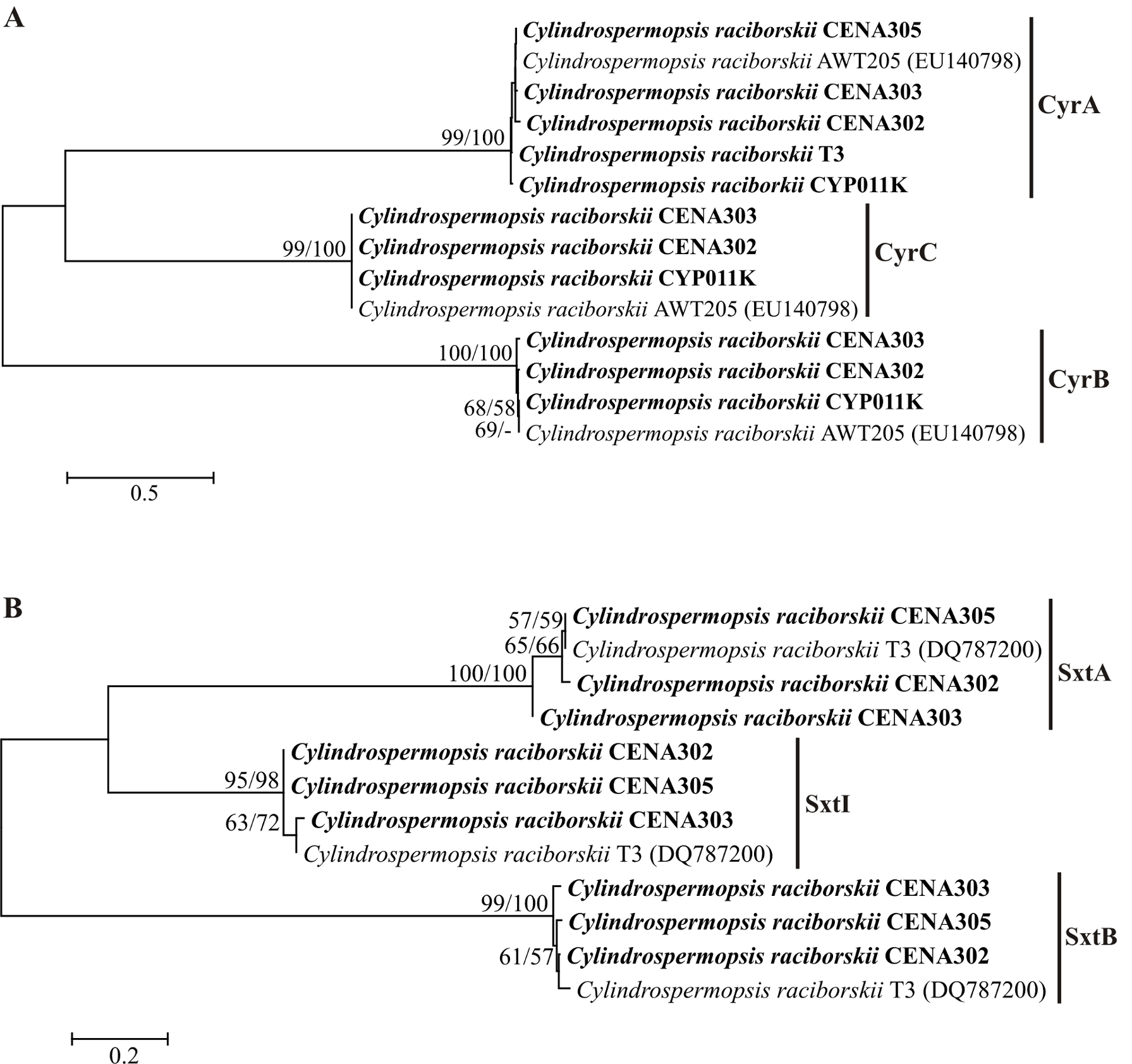

Supplement: Figure S2 — Maximum likelihood phylogenetic trees of Cyr (A) and Sxt (B) amino acids sequences. The C. raciborskii strains used in this study are shown in bold. Bootstrap test (1,000 resamplings) was performed and values >50% for ML and NJ analyses are shown over the nodes. (TIF) [file pone.0074238.s002.tif]
