# Supplementary material for: Cylindrospermopsin and Saxitoxin Synthetase Genes in Cylindrospermopsis raciborskii Strains from Brazilian Freshwater
Source: PLoS One. 2013 Aug 28;8(8):e74238. doi: 10.1371/journal.pone.0074238 (PMC3756036; doi:10.1371/journal.pone.0074238)
Supplement: Table S1 — PCR primer sequences used in this study. (DOCX) [file pone.0074238.s003.docx]

Table S1. PCR primer sequences used in this study.

| **Primer** | **Sequence (5’- 3’)** | **Gene** | **Gene product** | **Size (bp)** | **Reference** |
| --- | --- | --- | --- | --- | --- |
| 27F1  1494Rc | AGAGTTTGATCCTGGCTCAG  TACGGCTACCTTGTTACGAC | ***rrn*** | 16S rRNA | 1400 | Neilan et al., 1997 |
| SXTA4-F  SXTA4-R | GGACTCGGCTTGTTGCTTC  CCAGACAGCACGCTTCATAA | ***sxtA*4** | AONS, class I and II aminotransferase | 200 | This work |
| OCT-F  OCT-R | TGCCGTTTTGTGCTTAGATG  GGACGGAAGGACTCACGATA | ***sxtI*** | *O*-carbamoyl-  transferase | 923 | This work |
| SXTB-F  SXTB-R | TTTGTAGGRCAGGCACTT  ATCATCGGTATCATCGGTAG | ***sxtB*** | Cytidine deaminase | 400 | This work |
| CYLAT-F  CYLAT-R | ATTGTAAATAGCTGGAATGAGTGG  TTAGGGAAGTAATCTTCACAG | ***cyrA*** | Amidino-transferase | 1105-1179 | Kellmann et al., 2006 |
| CPS-F  CPS-R | AGTATATGTTGCGGGACTCG  CCCGCCAAGACAGAGGGTAG | ***cyrB*** | Adenylation domain (NRPS) | 478 | Kellmann et al., 2006 |
| A205PK-F  A205PK-R | AATGACAGAGACTTGTGCGGGG  TTATCGGTATTGGTGGTAGCAACT | ***cyrC*** | Ketosynthase domain (PKS) | 514 | Kellmann et al., 2006 |
| cyrJ-F  cyrJ-R | TTCTCTCCTTTCCCTATCTCTTTATCT  GCTACGGTGCTGTACCAAGGGGC | ***cyrJ*** | Putative sulfotransferase | 536 | Mazmouz et al., 2010 |

AONS: 8-amino-7-oxononanoate synthase; NRPS: Nonribosomal Peptide Synthetase; PKS: Polyketide Synthase
