# Supplementary material for: Cylindrospermopsin and Saxitoxin Synthetase Genes in Cylindrospermopsis raciborskii Strains from Brazilian Freshwater
Source: PLoS One. 2013 Aug 28;8(8):e74238. doi: 10.1371/journal.pone.0074238 (PMC3756036; doi:10.1371/journal.pone.0074238)
Supplement: Table S2 — Percentage of identities of cyr nucleotide sequences of CYN-non-producing Brazilian strains of C. raciborskii with other sequences from CYN-producing cyanobacterial strains. (DOCX) [file pone.0074238.s004.docx]

Table S2. Percentage of identities of partial *cyr* gene sequences of CYN-non-producing Brazilian strains of *C. raciborskii* with other sequences from CYN-producing cyanobacterial strains.

| ***cyrA* identities (%)** | | | | |
| --- | --- | --- | --- | --- |
| **Strain (Acession Number)** | **CENA302** | **CENA303** | **CENA305** | **T3** |
| *C. raciborskii* CYP011K | 99.1 | 99.2 | 98.8 | 99.3 |
| *C. raciborskii* AWT205 (EU140798) | 99.5 | 99.6 | 99.2 | 99.5 |
| *C. raciborskii* CS-505 (NZ_ACYA01000027) | 99.5 | 99.6 | 99.2 | 99.5 |
| *R*. *curvata* CHAB1150 (JN873921) | 99.2 | 99.3 | 98.8 | 99.2 |
| *Aphanizomenon* sp. 10E6 (GQ385961) | 98.8 | 98.9 | 98.5 | 98.8 |
| *Aph. ovalisporum* (AF395828) | 95.9 | 95.9 | 95.5 | 95.9 |
| *Oscillatoria* sp. PCC 6506 (FJ418586) | 87.0 | 87.1 | 86.8 | 87.1 |
| ***cyrB* identities (%)** | | | | |
| **Strain (Acession Number)** | **CENA302** | | **CENA303** | |
| *C. raciborskii* CYP011K | 99.6 | | 99.4 | |
| *C. raciborskii* AWT205 (EU140798) | 99.6 | | 99.4 | |
| *C. raciborskii* CS-505 (NZ_ACYA01000027) | 99.6 | | 99.4 | |
| *R*. *curvata* CHAB1150 (JN873921) | 99.6 | | 99.4 | |
| *Aphanizomenon* sp. 10E6 (GQ385961) | 99.6 | | 99.4 | |
| *Aph. ovalisporum* (AF395828) | 96.2 | | 96.0 | |
| *Oscillatoria* sp. PCC 6506 (FJ418586) | 85.1 | | 84.6 | |
| ***cyrC* identities (%)** | | | | |
| **Strain (Acession Number)** | **CENA302** | | **CENA303** | |
| *C. raciborskii* CYP011K | 100 | | 99.8 | |
| *C. raciborskii* AWT205 (EU140798) | 100 | | 99.8 | |
| *C. raciborskii* CS-505 (NZ_ACYA01000027) | 100 | | 99.8 | |
| *R*. *curvata* CHAB1150 (JN873921) | 99.8 | | 100 | |
| *Aphanizomenon* sp. 10E6 (GQ385961) | 99.6 | | 99.8 | |
| *Aph. ovalisporum* (AF395828) | 99.2 | | 99.4 | |
| *Oscillatoria* sp. PCC 6506 (FJ418586) | 91.5 | | 91.2 | |
