# Supplementary material for: Cylindrospermopsin and Saxitoxin Synthetase Genes in Cylindrospermopsis raciborskii Strains from Brazilian Freshwater
Source: PLoS One. 2013 Aug 28;8(8):e74238. doi: 10.1371/journal.pone.0074238 (PMC3756036; doi:10.1371/journal.pone.0074238)
Supplement: Table S3 — Percentage of identities of sxt nucleotide sequences of C. raciborskii Brazilian strains with other sequences from STX-producing cyanobacterial strains. (DOCX) [file pone.0074238.s005.docx]

Table S3. Percentage of identities of partial *sxt* gene sequences of *C. raciborskii* Brazilian strains with other sequences from STX-producing cyanobacterial strains.

| ***sxtA4* identities (%)** | | | |
| --- | --- | --- | --- |
| **Strain (Acession Number)** | **CENA302** | **CENA303** | **CENA305** |
| *C. raciborskii* T3 (DQ787200) | 99.5 | 99.5 | 100 |
| *R. brookii* D9 (NZ_ACYB01000035) | 99.5 | 99.5 | 100 |
| *A. circinalis* AWQC131C (DQ787201) | 92.0 | 92.0 | 92.6 |
| *Aphanizomenon* sp. NH-5 (EU603710) | 92.0 | 92.0 | 92.6 |
| *L. wollei* Carmichael/Al (EU603711) | 90.0 | 90.0 | 90.6 |
| ***sxtB* identities (%)** | | | |
| **Strain (Acession Number)** | **CENA302** | **CENA303** | **CENA305** |
| *C. raciborskii* T3 (DQ787200) | 100 | 97.7 | 99.4 |
| *R. brookii* D9 (NZ_ACYB01000035) | 100 | 97.7 | 99.4 |
| *A. circinalis* AWQC131C (DQ787201) | 86.6 | 84.5 | 86.6 |
| *Aphanizomenon* sp. NH-5 (EU603710) | 84.7 | 82.6 | 84.7 |
| *L. wollei* Carmichael/Al (EU603711) | 86.8 | 85.1 | 86.8 |
| ***sxtI* identities (%)** | | | |
| **Strain (Acession Number)** | **CENA302** | **CENA303** | **CENA305** |
| *C. raciborskii* T3 (DQ787200) | 99.8 | 99.5 | 99.6 |
| *R. brookii* D9 (NZ_ACYB01000035) | 99.7 | 99.1 | 99.5 |
| *A. circinalis* AWQC131C (DQ787201) | 89.0 | 89.6 | 89.2 |
| *Aphanizomenon* sp. NH-5 (EU603710) | 89.5 | 89.1 | 89.6 |
| *L. wollei* Carmichael/Al (EU603711)* | – | – | – |

(*) truncated or inactive sequence (Kellmann et al., 2008b).
