# Supplementary material for: Cylindrospermopsin and Saxitoxin Synthetase Genes in Cylindrospermopsis raciborskii Strains from Brazilian Freshwater
Source: PLoS One. 2013 Aug 28;8(8):e74238. doi: 10.1371/journal.pone.0074238 (PMC3756036; doi:10.1371/journal.pone.0074238)
Supplement: Table S4 — The 16S rRNA gene sequences identities among the Brazilian C. raciborskii strains and other sequences of related cyanobacterial strains. (DOCX) [file pone.0074238.s006.docx]

| **Strain** | **Origin** | **16S rRNA sequence size (bp)** | **1** | **2** | **3** | **4** | **5** | **6** | **7** | **8** | **9** | **10** | **11** | **12** |
| --- | --- | --- | --- | --- | --- | --- | --- | --- | --- | --- | --- | --- | --- | --- |
| 1. *C. raciborskii* CENA302 | Brazil | 1,433 | - |  |  |  |  |  |  |  |  |  |  |  |
| 2. *C. raciborskii* CENA303 | Brazil | 1,412 | 100 | - |  |  |  |  |  |  |  |  |  |  |
| 3. *C. raciborskii* CENA305 | Brazil | 1,412 | 99.1 | 99.1 | - |  |  |  |  |  |  |  |  |  |
| 4. *C. raciborskii* T3 | Brazil | 1,412 | 99.4 | 99.4 | 99.8 | - |  |  |  |  |  |  |  |  |
| 5. *C. raciborskii* LEGE 051 | Brazil | 1,382 | 98.5 | 98.5 | 99.1 | 99.2 | - |  |  |  |  |  |  |  |
| 6. *C. raciborskii* CYP-011K | Australia | 1,412 | 99.2 | 99.2 | 99.4 | 99.6 | 98.8 | - |  |  |  |  |  |  |
| 7.  *C*. *raciborskii* AWT205 | Australia | 1,447 | 99.2 | 99.2 | 99.4 | 99.6 | 98.8 | 99.7 | - |  |  |  |  |  |
| 8.  *C*. *raciborskii* CS-505 | Australia | 1,444 | 99.4 | 99.4 | 99.5 | 99.7 | 99.1 | 99.8 | 99.8 | - |  |  |  |  |
| 9. *C. raciborskii* PMC98.14 | France | 1,435 | 99.4 | 99.4 | 99.5 | 99.7 | 98.9 | 99.7 | 99.7 | 99.9 | - |  |  |  |
| 10*. C. raciborskii* FAS-C1 | USA | 1,432 | 99.3 | 99.3 | 99.6 | 99.8 | 99.0 | 99.6 | 99.6 | 99.8 | 99.9 | - |  |  |
| 11. *Raphidiopsis brookii* D9 | Brazil | 1,481 | 99.3 | 99.3 | 99.7 | 99.8 | 99.3 | 99.5 | 99.5 | 99.0 | 99.6 | 99.7 | - |  |
| 12. *Aphanizomenon* sp. 10E6 | Germany | 1,040 | 94.4 | 94.4 | 94.4 | 94.6 | 94.4 | 94.3 | 94.3 | 94.5 | 94.4 | 94.4 | 94.4 | - |
| 13. *Oscillatoria* sp. PCC6806 | Unknow | 1,480 | 90.2 | 90.1 | 90.1 | 90.1 | 89.7 | 89.8 | 90.1 | 90.2 | 90.2 | 90.1 | 90.4 | 92.5 |

Table S4. The 16S rRNA gene sequences similarities among the Brazilian *C. raciborskii* strains and other sequences of related cyanobacterial strains.
